# Quality of life profiles and its association with predictors amongst Chinese older adults in nursing homes: a latent profile analysis

**DOI:** 10.1186/s12877-023-04456-2

**Published:** 2023-11-14

**Authors:** Chunqin Liu, Qing Luo, Dongyi Luo, Ying Zhou, Xue Feng, Zihan Wang, Jiajian Xiao, Qiulin Bi, Graeme Drummond Smith

**Affiliations:** 1 School of Nursing, Guangzhou Medical University, Guangzhou, Guangdong 510182 China; 2 School of Medicine, Jinggang Shan University, Jian, Jiangxi 343009 China; 3 School of Heath Industry, Guangdong Open University, Zhongshan, Guangdong 528499 China; 4 Finance Division of Guangzhou Medical University, Guangzhou, Guangdong 511436 China; 5 Guangzhou Songhe Nursing Home, Guangzhou, Guangdong 510250 China; 6 School of Health Sciences, Caritas Institute of Higher Education, Hong Kong SAR, 999077 China

**Keywords:** Quality of life, Older adults, Nursing home, Latent profile analysis, Gerontology

## Abstract

**Background:**

Recently developments in the field of positive psychology have provided new perspectives for understanding the connection between individual variation in Quality of life (QoL) and positive aspects of human potential, strengths, and resources, commanding increasing attention. This study aimed to examine self-reported quality of life (QoL) profiles and the association of QoL profiles with positive psychosocial characteristics in Chinese older adults.

**Methods:**

A convenient sample of 354 older adults in nursing homes was recruited from Guangdong Province, China, between November 2020 and January 2021. Latent Profile Analysis (LPA) was conducted to explore QoL profiles using the four WHOQOL-BREF domains as input variables. Multinomial logistic regression was performed to explore the association between latent profiles and predictors.

**Results:**

LPA identified three latent QoL profiles: “low QoL with poor psychological health” (18.1%), “moderate QoL” (46.0%) and “high QoL” (35.9%). Frequency of weekly activity, optimism, gratitude, and social support were associated with the increased likelihood of belonging to the moderate-to-high QoL classes. Furthermore, Class 2 (moderate QoL group, reference) was compared with Class3 (high QoL group), higher frequency of weekly physical activity and spending more time on physical activity exhibited higher odds of belonging to high QoL class.

**Conclusion:**

Using the domains of the WHOQOL-BREF scale, the QoL profiles Chinese older adults can be identified. We found that psychosocial variables and demographic characteristic, including lower level of optimism and gratitude, lack of social support, low frequency of physical activity, and shorter activity duration time, heighten the risk for lower levels of QoL. Identifying classification may help focus on those at elevated risk for poor QoL and for developing tailored QoL improvement programs.

**Supplementary Information:**

The online version contains supplementary material available at 10.1186/s12877-023-04456-2.

## Background

Globally, aging has become an increasingly urgent issue and a significant challenge facing public healthcare [1]. China has one of the world’s largest ageing populations, the proportion of the population aged 65 or older had reached 14.2% in 2021, representing 200.6 million people, and this number is projected increase to 400 million by 2050 [2]. Inevitably, with greater longevity the number of chronic age-related diseases will increased. The existing disconnected and fragmented healthcare may fail to meet the demands of the ageing population, such as lack of care coordination and the uneven distribution of health services, resulting in enormous costs and burdens on the system [3]. It is estimated that older people will consume 75% of health care resources by 2030 [4]. How to fulfill healthy ageing has become a key public health challenge with the escalation of population aging. Quality of life (QoL) is one concept that has been identified as a critical aspect for healthy ageing and health promotion in old age [5].

QoL is a broad multidimensional construct assessing individuals’ perception of overall wellbeing and life, involving physical health, psychological state, social functioning, and environment [6]. Lower levels of QoL are strongly associated with adverse health outcomes. For example, poorer QoL in later life is significantly predictive of a higher frequency of hospitalization, reduced physical independence and psychosocial well-being level and, notably, increased socioeconomic burden and mortality amongst older adults [7]. Conversely, those negative outcomes have a deteriorate effect upon QoL, creating a vicious cycle that ultimately may result in decreased life expectancy [8]. On the other hand, ageing can be accompanied with the onset of comorbid diagnoses, disability, changes in lifestyle behavior, and the potential loss of independence, all of which lead to poorer QoL [8, 9]. Given the increase in life expectancy and the importance role of QoL in older people’s, maintaining and improving QoL at an advanced age has become an increasingly important nursing consideration.

Regarding QoL, literature has mainly focused on the influencing factors of QoL in older age, pointing to a dynamic interaction amongst these factors. Specifically, QoL can be affected in a complex manner by demographic and health characteristics, psychological states, social engagement, and living conditions [5, 10]. For instance, having multiple chronic diseases, cognitive and physical impairments, polypharmacy, and psychological morbidity may have a negative relationship with QoL [11, 12]. Whilst, independence in daily living life, engaging in physical exercise and other healthy lifestyle behaviors are positive predictors for good QoL [13]. In addition, there is a recognized link between social support and QoL in older adults, that is, the quality and quantity of an individual’ social support interactions can be positively related to both their health status and QoL [10, 14].

Recently developments in the field of positive psychology have provided new perspectives for understanding the connection between individual variation in QoL and positive aspects of human potential, strengths, and resources, commanding increasing attention [15]. Several positive psychological constructs have been identified to be beneficial in the field of healthy ageing [16, 17], including optimism, which can be defined as the tendency to expect positive outcomes across a variety of life domain [18]. Numerous studies have shown that optimism is a positively correlated with well-being and physical health in older people [19, 20]. Optimists are capable of adapting to and dealing with the negative impacts of ageing, reporting more positive affect and life satisfaction, and less negative affect [21]. Similarly, a graded and independent positive relationship has been reported between optimism and objective indicators of QoL (e.g., mortality) in older people [22, 23].

Gratitude, another relevant positive psychological trait in older people, it can be defined as “a generalized orientation toward recognizing and appreciating the positive in the world” [24]. Given the robust association between gratitude and multiple domains of physical and mental well-being, this construct may represent a relevant and meaningful process to positively affect QoL [24]. In addition, gratitude has been shown to be an important and independent contributor to bolster QoL, even in people struggling with adversity, such as chronic illness [25, 26]. Despite this, the association between gratitude and QoL in older people, particularly those residing in nursing residential care homes, has received little attention.

Previous studies have focused on the relationship between the total QoL scores of older adults and individual variables. However, these studies have mostly adopted a traditional variable-centered approach (e.g., regression analysis) [27, 28], ignoring the multidimensional features of QoL and the heterogeneity of sample and limiting the ability to precision intervene. Latent profile analysis (LPA), a person-centered modeling approach that attempts to identify and characterize unobserved subpopulations of multiple observed variables that recur among individuals, thereby classify heterogeneous samples into more meaningful subgroups [29, 30]. Importantly, this method allows for the identification of populations most in need of intervention and identifies where there is a need for intervention in different domains [31]. In recent years, LPA has been applied to examine QoL in older adulthood, confirming four distinct health-related QoL profiles (stable type, physical disability type, emotional disability type, and crisis type) using the European Quality of Life-5 Dimensions Questionnaire (EQ-5D) scale among community-dwelling older Korean adults [32]. Following a similar approach, Băjenaru et al. [33] identified three subgroups using the World Health Organization Quality of Life-Brief (WHOQOL-BREF) instrument in older Romanian patients, low and very low QoL, moderate QoL, and high QoL. Therefore, the application of LPA can give the potential to identify homogeneous QoL subgroups, allowing us to better understand patterns across older population and the differences between patterns.

The past few years have seen vigorous development of China’s institutional care model. According to the 2022 National Economic and Social Development Statistical Bulletin issued by the National Bureau of Statistics [34], China has approximately 40,000 nursing homes with 8.223 million nursing beds and 34 beds per thousand older adults. Due to the late development of China’s nursing home system, most nursing care institutions mainly focus on life care and lack professional medical and healthcare services, and these institutions cannot fully meet the healthcare needs of care home residents. Previous studies have shown that nursing home residents face a higher risk of poor QoL than community-dwelling older people because of poor health status, high level of dependency, absence of physical activities, and social isolation [35]. Applying an appropriate statistical approach to focus on assessing the QoL of older adults in these institutions is pivotal to improved patient care. However, to the best of our knowledge, no study has applied LPA to study QoL in older adults in Chinese nursing homes. More importantly, there is scant information concerning the contribution of positive psychological constructs (e.g., gratitude) to the QoL and its specific subgroups in residential care setting. Having a greater understanding of these psychological characteristics of QoL latent subgroups is crucial for developing resources in a targeted manner to improve QoL amongst older people.

The aim of this study is to classify patterns of QoL in Chinese older adults living in nursing homes using the LPA method, and determined whether sociodemographic factors (e.g., age), health-related factors (e.g., physical health status), and positive psychosocial factors (e.g., gratitude) are associated with a specific profile membership of QoL. We anticipate that the finding of this research may help to develop tailored interventions based upon identified patterns, particularly for those with poorer level of QoL, to enhance QoL in this population.

## Methods

### Design

This was a cross-sectional study that focused on older adults sampled from three nursing homes in Guangzhou, China that had long-term cooperative relationships with the study team.

### Participants

Detailed data collection methods have been described in a previous study [36]. Briefly, two trained nursing researchers conducted in-person interviews using structured questionnaires to investigate the level of subjective well-being and positive psychosocial factors, including optimism, gratitude, and other variables in residents of nursing homes from November 2020 to January 2021. They also investigated the quality of life in those older adults. All participants were ≥ 60 years of age, had lived at least one month in a nursing home, had no cognitive impairment, and consented to participate. Participants were excluded if they had significant communication deficits or acute or end-stage medical conditions. All three nursing homes were located in urban districts of Guangzhou, were privately owned, had a similar size (more than 300 beds), and belonged to the medical-nursing combined model. Furthermore, they provided diverse types of activities, such as playing chess, singing, and dancing, to meet the daily life needs of older adults. As such, there were no significant differences between the facilities in terms of healthcare models or activity types (Supplementary Table S1). Ethical approval for this study was obtained from Guangzhou Medical University Institutional Review Board with the number of 202,042,009, after receiving information about the study the respondents and/or their legal guardians signed the written informed consent to participate in our study, the voluntary nature of the study and their data anonymity and confidentiality were strictly assured. The sample size was calculated using Kendall’s sample size estimation method, ensuring it would be 5–10 times that of the number of variables [37]; the number of variables in this study was 22 and so the sample size was estimated to be 110–220. Considering the possibility of invalid questionnaires, the sample size was expanded by 20%, for a final sample size of 132–264. In total, we enrolled 380 nursing home residents and sixteen responses were excluded due to the missing or invalid data, 354 participants were included for final analysis (response rate 93.15%).

### Measurement outcomes

#### Demographic, health-related, and clinical characteristics of participants

Demographic information was collected from participants including age, gender, marital status, education, monthly pension, number of children and weekly visit by their children. Health-related variables included smoking(yes/no), drinking status(yes/no), number of times and the duration of physical activity within the previous seven days. Clinical characteristics included the presence of chronic diseases, medication regime, and number of hospitalizations within the previous year. In our study, the number of chronic diseases was defined as the sum of the presence of self-reported cardiovascular disease, cerebrovascular disease, respiratory system diseases, endocrine, nutritional, and metabolic diseases, cancer, and other diseases. The medication regime was assessed via one question, “Have you taken medications currently?”. If the older adult responded “yes”, the next question would be asked, “How many medications did you take?”. Polypharmacy was termed as when individual has taken five prescriptions drugs or more daily based upon the previously published study [38]. Number of hospitalizations was investigated by one question, “Have you been hospitalized in the past year?”.

#### Quality of life

The World Health Organization Quality of Life-Brief (WHOQOL-BREF) was used to assess individuals’ levels of QoL [6, 39]. The measurement includes 26 items, and 24 of these items are divided into the following four domains: physical health (seven items; e.g., “How well are you able to get around”), psychological health (six items; e.g., “How much do you enjoy life”), social relationships (three items; e.g., “How satisfied are you with your personal relationships”), and environmental health (eight items; e.g., “How safe do you feel in your daily life”).Two items assess the perception of overall quality of life and general health. All items are rated on a 5-point scale, and raw domain scores via a formula were converted to a scale ranging from 0 to 100, with higher scores denoting higher QoL [40]. In present study, Cronbach’s alpha was 0.94, 0.85, 0.85, 0.70 and 0.82 for the total WHOQOL-BREF scale, the physical health, psychological health, social relationships, and environmental health, respectively.

#### Optimism

The Life Orientation Test-Revised (LOT-R) scale was composed of 10 items, including three items to assess optimism, three items to assess pessimism, and four filler items [41, 42]. Sample item was “In uncertain times, I usually expect the best”. Participants indicated level of agreement on a 5-point scale, from 0 (strongly disagree) to 4(strongly agree); Optimism and pessimism scores were calculated by adding the corresponding three items, with a score range of 0 to 12. Total scores were summed up the optimism score and the inverted pessimism score, with a higher score suggesting greater sense of optimism. The Cronbach’s alpha coefficient for the LOT-R was 0.82.

#### Gratitude

The Gratitude questionnaire-six-item form (GQ-6) is a unidimensional scale with six items designed to assess the level of gratitude (e.g., “I have so much in life to be thankful for”) [43, 44]. Each item was responded to using a 7-point scale (1 = strongly disagree, 7 = strongly agree). Higher scores represented higher levels of gratitude. The scale demonstrated satisfactory reliability in current study with Cronbach’s alpha of 0.92.

#### Social support

The Perceived Social Support Scale (PSSS) included 12 items and was used to quantify the level of social support [45, 46]. It assessed the degree to which older adults perceive their support from family, friend, and significant others (e.g., I get the emotional help and support from my family when in need). Participants responded to the items on a 7-point scale from 1 (very strongly disagree) to 7 (very strongly agree). Higher values indicated greater sense of perceived social support. The Cronbach’s alpha coefficient in the study was 0.93.

### Data analysis

Descriptive statistics and bivariate correlations were analyzed using SPSS 26.0. Mplus 8.3 software was used to conduct the LPA and identify the QoL subgroups based on the four WHOQOL-BREF domains. A series of model fit indices were used to determine the optimal number of latent subgroups, including Akaike information criterion (AIC), Bayesian information criterion (BIC), sample-size-adjusted BIC (ABIC), and the Vuong-Lo-Mendell-Rubin likelihood ratio test (VLMR-LRT), bootstrapped likelihood ratio test (BLRT) and Entropy. Lower values in the AIC, BIC and ABIC represented a better model fit. LMR and BLRT compared the model fit improvements between two neighboring models, and a significant *P*-value suggested that the k-class model fits the data better than the k-1-class model [47]. Entropy was used to determine the classification quality, with a value closer to 1 indicating a better separation of the classes [48]. Additionally, the posterior probability of classes was used to further validate the classifications, with a value ≥ 0.80 representing good discriminability [49]. Once the profiles were identified, each profile was named to best describe its characteristics and to differentiate it from other profiles. Next, an ANOVA was conducted to test the differences in the QoL subscales between the three classes. Subsequently, differences in demographic, clinical and psychosocial variables were analyzed using the chi-square and ANOVA tests for the QoL potential subtypes. Multinomial logistic regression analysis was conducted to explore the association between the identified QoL profiles and independent variables. A two-tailed *P* < 0.05 was considered as statistically significant.

## Results

### Descriptive statistics and correlations

A total of 354 older adults were analyzed in the study, with a mean (SD) age of 86.01(6.48) years, (range: 61–108 years). Among them, 264(74.6%) were females and 90(254%) were males. The majority were widowed (222, 62.7%) or married (118, 33.3%). Nearly half of the residents (48.6%) had an elementary school or below education; 61.3% reported having a monthly pension income of ≤ 5,000 Chinese yuan, 52.5% had 3 + children; and 79.1% of residents had child visits per week. Nearly all were non-smokers (96.9%) or non-drinkers (97.7%); 74.6% reported performing at least one type of physical activity at least seven times per week, and 69.8% reported at least one hour of each activity. In terms of clinical characteristics, the mean number of chronic diseases was 2.01 (SD 0.77; range 0–6) and 94.1% of residents reported having at least one chronic disease; 48% presented polypharmacy, and 20.3% reported hospitalizations in the past year. From the four QoL subscales, the environmental health domain had the highest mean score of 61.9(12.3) and the social relationships domain had the lowest mean score of 58.1(11.9). In terms of psychosocial variables, the mean score of optimism, gratitude, and social support were 17.3(3.2), 24.9(7.2), and 56.0(9.5), respectively. The bivariate correlations among the study variables are presented in Supplemental Table S2. QoL subscales showed small correlations with optimism, gratitude, and social support (all correlations were below 0.50).

### Latent profile analysis for QoL

The model fit statistics for 1–4 latent profile models were presented in Table 1. With an increase in the number of latent profiles, the AIC, BIC, and ABIC gradually decreased, and the BLRT showed significant results in comparisons, which are all the models with k and k-1class. Therefore, these values did not suggest any latent solution. Although the 4-class model had the highest entropy, the *P*-value of the VLMR-LRT was 0.264, suggesting that the 4-class model was rejected. The 3-class model emerged a new class with clinically relevant patterns of item-response probabilities when compared with the 2-class solution. Moreover, classification quality, based on the average latent profile probabilities for most likely class membership, suggested that the 3-classes had a good discriminability and reliable classification: 0.918 for latent class 1; 0.925 for latent class 2; 0.924 for latent class 3. As such, the 3‐class model was chosen for the following analysis.

**Table 1:** Model fit indices for the compared latent profiles(n = 354)

Model	AIC	BIC	ABIC	VLMR *P*-value	BLRT-LRT *P*-value	Entropy	Smallest class (%)	Posterior probability			
								1	2	3	4
1-class	11376.209	11407.164	11381.784	NA	NA	NA	NA	NA			
2-class	10821.362	10871.662	10830.421	< 0.001	< 0.001	0.832	41.808	0.954	0.947		
**3-class**	**10605.241**	**10674.889**	**10617.785**	**0.047**	**< 0.001**	**0.835**	18.079	**0.918**	**0.925**	**0.924**	
4-class	10519.637	10608.631	10535.665	0.264	< 0.001	0.854	5.932	0.900	0.910	0.783	0.947

Note: The model solution chosen is bolded. NA = not applicable; AIC = Akaike Information Criteria; BIC = Bayesian Information Criteria; ABIC = Adjusted Bayesian Information Criteria; VLMR-LRT = Vuong-Lo-Mendell-Rubin likelihood ratio test; BLRT = Bootstrapped Likelihood Ratio Test; Smallest class (%) = Smallest percentage of each class

**Fig. 1:**
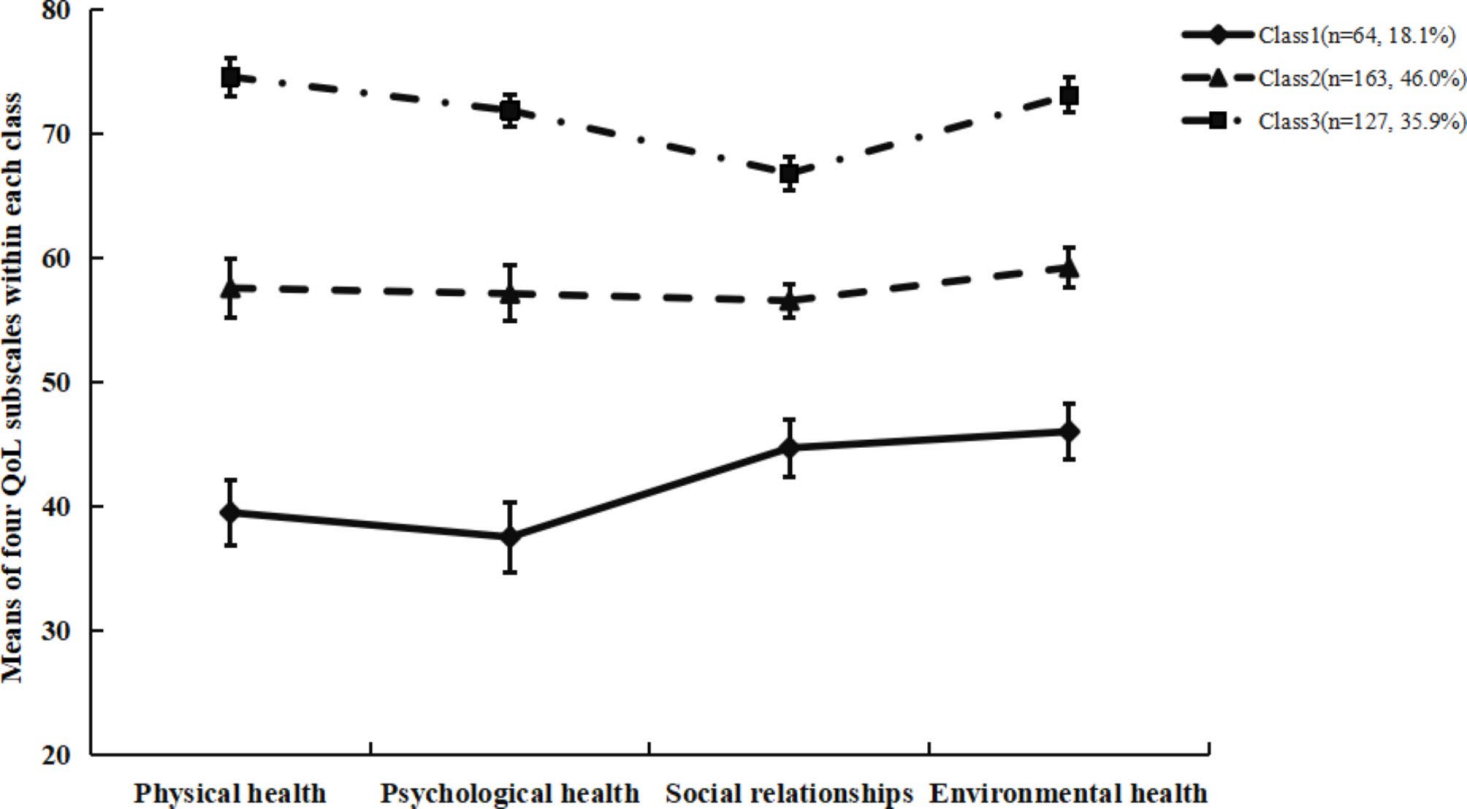
QoL subscales for the latent profile classes. Note: Class1 = Low QoL with poor psychological health, Class2 = Moderate QoL, Class3 = High QoL. Whiskers indicate the standard error of the mean

Figure 1 illustrated the three classes. Class 1 consisted of 64 (18.1%) older adults and was characterized by the lowest QoL scores for all four subscales compared with the other group, particularly on psychological domain, and therefore was named ‘low quality of life with poor psychological health’ subgroup. Class 2(n = 163, 46.0%) was closed to the average QoL scores on each subscale and was labelled as ‘moderate quality of life’ subgroup. Class 3(n = 127, 35.9%) was characterized by a high QoL score and higher than average QoL scores across all subscales and was considered as ‘high quality of life’ subgroup.

The ANOVA and Bonferroni post-hoc tests indicated that QoL subscales scores differed in all three classes (*P* < 0.05), with psychological health exhibited the largest effect size (η^2^ = 0.749). Specifically, psychological health differentiates the QoL classes from each other the most, suggesting that it is the most important variable for the classification. Furthermore, as all dimensions of QoL differ between the classes in the same direction (i.e., those lowest in one dimension were likely to be the lowest in the other three), it supported the interpretability of the classifications by LPA (See Table 2).

**Table 2:** Differences of QoL subscales scores among three different latent classes

Variables	Class1 *n* (%) 64 (18.1)	Class2 *n* (%) 163(46.0)	Class3 *n* (%) 127 (35.9)	*P*	*η2*	Bonferroni post-hoc test
QoL subscales scores: M(SD)						
Physical health	39.2(9.8) ^a^	57.5(8.7) ^b^	75.1(8.0) ^c^	< 0.001	0.685	a < b, c; b < c
Psychological health	37.2(8.3) ^a^	57.2(7.0) ^b^	72.2(6.8) ^c^	< 0.001	0.749	a < b, c; b < c
Social relationships	44.9(9.8) ^a^	56.5(8.3) ^b^	66.9(9.6) ^c^	< 0.001	0.424	a < b, c; b < c
Environmental health	45.7(7.5) ^a^	59.2(6.9) ^b^	73.4(7.6) ^c^	< 0.001	0.652	a < b, c; b < c

Note: Class1 = Low QoL with poor psychological health, Class2 = Moderate QoL, Class3 = High QoL.

a, b, c showed the results of the Bonferroni post-hoc tests and there is a statistically significant between two. e.g. for physical ‘a < b, c’ means that class 1 has a mean score that is lower than class 2 and 3.

### Comparison of demographic and psychosocial variables in each latent profile

Table 3 outlined the comparison of demographic and psychosocial variables in different profiles. Among these variables, gender, education, pension, weekly frequency of physical activity, average hours spent per time on physical activity, number of chronic diseases, optimism, gratitude, and social support were different among the three QoL classes (all *P* < 0.05). Additionally, there were no significant differences with respect to age, marital status, number of children, has child visits per week, smoking, drinking, polypharmacy, and hospitalizations in the past year among the three QoL classes (all *P* > 0.05).

**Table 3:** Profiles of QoL in demographic and psychosocial characteristics among older adults(n = 354)

Variables		Class1 *n* (%) 64(18.1)	Class2 *n* (%) 163(46.0)	Class3 *n* (%) 127 (35.9)	*χ* ^*2*^ */F*	*P*
Age(years) M(SD)		87.5(5.5)	86.0(6.5)	85.4(6.5)	2.282	0.104
Gender	Female Male	55(85.9) 9(14.1)	114(69.9) 49(30.1)	95(74.8) 32(25.2)	6.209	**0.045**
Marital status	Married Single/divorced/ widowed	16(25.0) 48(75.0)	64(39.3) 99(60.7)	38(29.9) 89(70.1)	5.292	0.073
Education	Elementary school and under	37(57.8)	84(51.5)	51(40.2)	13.918	**0.031**
	Middle school	18(13.7)	31(19.0)	27(21.3)		
	High school	6(9.4)	29(17.8)	32(25.2)		
	College and above	3(4.7)	19(18.0)	17(13.4)		
Pension (RMB)	≤ 5000	49(76.6)	101(62.0)	67(52.8)	12.243	**0.016**
	5001–9000	13(20.3)	47(28.8)	41(36.2)		
	> 9000	2(3.1)	15(9.2)	19(15.0)		
Number of children	≤ 2	35(54.7)	66(40.5)	67(52.8)	5.944	0.051
	3 and above	29(45.3)	97(59.5)	60(47.2)		
Has child visits per week	No	18(28.1)	36(22.1)	20(15.7)	4.198	0.123
	Yes	46(71.9)	127(77.9)	107(84.3)		
Smoking	No	64(100.0)	158(96.9)	121(95.3)	3.156	0.206
	Yes	0(0.0)	5(3.1)	6(4.7)		
Drinking	No	63(98.4)	162(99.4)	121(95.3)	5.633	0.060
	Yes	1(1.6)	1(3.7)	6(4.7)		
Activity frequency	< 7 times/week	41(64.1)	42(25.8)	7(5.5)	76.963	**< 0.001**
	≥ 7 times/week	23(35.9)	121(74.2)	120(94.5)		
Duration	< 1 hour/session	42(65.6)	49(30.1)	16(12.6)	56.740	**< 0.001**
	≥ 1 hour/session	22(3.4)	114(69.9)	117(87.4)		
Number of chronic diseases	≤ 2	44(68.8)	126 (77.3)	107(84.3)	6.168	**0.046**
	3 and above	20(31.3)	37(22.7)	20(15.7)		
Polypharmacy	No	30(46.9)	83(50.9)	71(55.9)	1.526	0.466
	Yes	34(53.1)	80(49.1)	56(44.1)		
Hospitalizations in the past year	No	52(81.3)	127(77.9)	103(81.8)	56.740	0.752
	Yes	12(18.8)	36(22.1)	24(18.9)		
Mean (SD)						
Optimism		14.2(3.6)	17.5(2.5)	18.8(2.7)	57.701	**< 0.001**
Gratitude		19.7(6.8)	24.5(6.7)	28.1(6.2)	35.501	**< 0.001**
Social support		48.6(8.5)	55.1(8.1)	61.0(8.8)	47.525	**< 0.001**

Note: RMB = Renminbi, Class1 = Low QoL with poor psychological health, Class2 = Moderate QoL, Class3 = High QoL

### Association of QoL profiles with demographic and psychosocial factors

We used multinomial logistic regression to examine the association of the QoL profiles with demographic and psychosocial factors. Only variables that were significantly associated with QoL in the univariate analysis were entered as the independent variables in the logistic regression analysis.

**Fig. 2:**
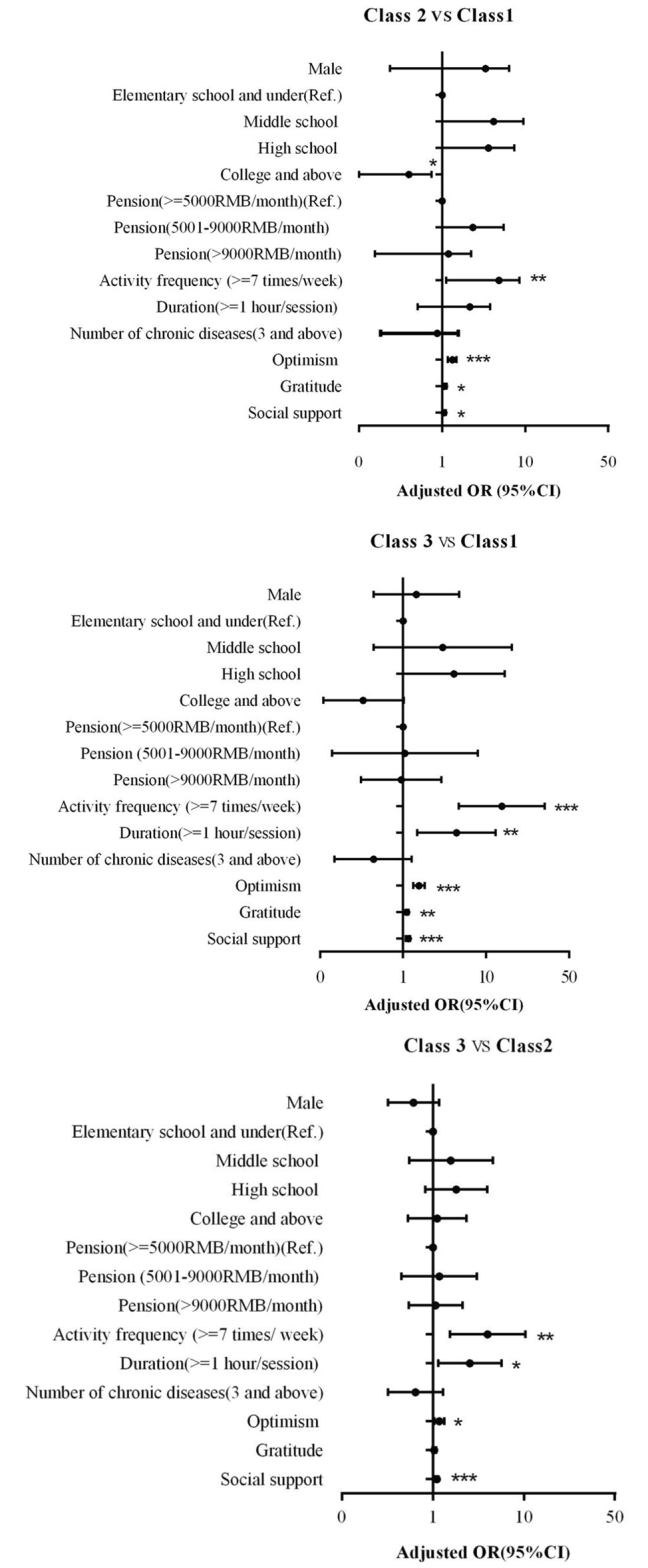
Multinomial logistic regressions showing the association of demographics, optimism, gratitude, and social support with Classes 2–3, compared with Class 1 and Class 2. The black filled circle represents the odds ratio (OR) and the horizontal line represents the 95% confidence interval (CI), ^***^p < 0.001 ^**^p < 0.01 ^*^p < 0.05. Class1 = Low QoL with poor psychological health, Class2 = Moderate QoL, Class3 = High QoL.

First, Class 1(low QoL with poor psychological health group, as the reference class) was compared with the other classes (Fig. 2). Weekly frequency of physical activity (OR = 3.88–15.47), optimism (OR = 1.32–1.55), gratitude (OR = 1.07–1.10), and social support (OR = 1.05–1.15) were associated with the increased likelihood of belonging to the moderate-to-high QoL classes. Additionally, the probability of belonging to the low QoL with poor psychological health subgroup was associated with a college degree or higher (OR = 0.29) as compared with their respective reference groups. The probability of belonging to the high QoL group was higher for the average time spent on physical activity more than 1 h (OR = 4.40) than for less than 1 h.

Second, Class 2(moderate QoL group, reference) was compared with Class3(high QoL group). Higher weekly frequency activity and longer average hours spent on activity exhibited higher odds of belonging to high QoL class (OR = 3.99; OR = 2.54). Also, the high QoL group had a high level of optimism (OR = 1.18) and social support (OR = 1.10).

## Discussion

The current study is the first to identify the heterogeneity of QoL and examined the variables associated with the latent subtypes via the WHOQOL-BREF, using LPA in Chinese older nursing homes residents. Two main findings emerged from our study. Firstly, three distinct profiles of QoL were identified, and these were named ‘low QoL with poor psychological mental’, ‘moderate QoL’, and ‘high QoL’. Secondly, six mainly associated factors of QoL profile were determined. A high frequency of weekly physical activity, increased time spent on activity, greater levels of optimism, gratitude, and social support were found to be associated with moderate-high QoL profiles. These findings allow the identification of predictors, which may facilitate with the development of tailormade-interventions to improve QoL of older adults.

Of the three profiles identified, the largest was the moderate QoL subgroup, to which 46.0% of older adults belonged, it was characterized by a moderate level of all WHOQOL-BREF domains (e.g., physical health, psychological health, social relationships, and environmental health). The second largest profile was the high QoL subgroup, accounting for 35.9% of older adults, these participants reported the highest level of QoL. The third profile (18.1% of older adulthood) was named low QoL with poor psychological health, as it was characterized by a low level of all QoL areas, particularly for psychological aspect. These results indicated that older adults’ perceptions of QoL varied, exhibiting individual differences. Among these groups, older adults in the low QoL with poor psychological health profile require the most attention. Thus, it is imperative to develop tailored interventions specifically targeting this population group, particularly in the realm of psychological health, so as to improve their QoL and promote healthy aging. Our findings are partially consistent with one recent Romanian study on variable-centered research of QoL, showing that the existence of three distinct QoL profiles based on WHOQOL-BREF four domains in older patients. Specifically, they found 63.3% of older people classified in the moderate QoL subgroup, 28.3% in the low QoL with poor psychological health subgroup, and 8.4% in the high QoL subgroup [33]. This discrepancy with our findings may be due to differences in sample characteristics, culture, or geographical region. Additionally, our study has revealed that the importance of psychological health in classifying the QoL among older adults. Consequently, within the different subscales of QoL, psychological health emerges as a crucial aspect that needs significant attention in our study. This finding suggests a potential necessity to customize interventions based on older adults’ psychological health condition.

The higher frequency of weekly physical activity and longer average time spent on activity, increase the likelihood of belonging to the moderate-high QoL group. Physical activity is considered a vital intervention for increasing overall well-being and quality of life in older adults [13, 50], and the frequency and duration of weekly physical activities appear to be important. The WHO evidence-based guidelines for physical activity amongst older adulthood clearly stipulates that at older adults require at least 150 to 300 min of moderate-intensity, or 75 to 150 min of vigorous-intensity physical activity every week to maintain health [51]. In addition, evidence suggests that increasing physical activity to recommended levels (moderate-to-vigorous activity performed in bouts of at least 10 min) can help prevent and reduce the prevalence of non-communicable diseases among older adults [52]. Studies have demonstrated that several types of exercise such as Yoga, Qigong, and Tai Chi all generated a positive effect on QoL of older people [53]. However, the level of physical activity among nursing home residents is generally low [54], possibly due to their inadequate awareness of activity and relatively poor physical condition. Because of this, institutional-level awareness programs and the provision of fitness facilities are required to motivate nursing home residents to engage in daily physical activity. Considering that the level of physical activity that care home residents can engage in may depend on their level of mobility, targeted physical activities should be developed to offer appropriate exercises based on the capabilities of individual residents. One unanticipated finding of this study was that older people with a college degree or higher had a higher likelihood of having poor QoL. This finding seems to correlate with the different perceptions of QoL in highly educated residents, attributed to their different attitudes, expectations, and coping strategies; this educated group generally has higher expectations, may be more critical of nursing home care, may be less willing to accept their circumstances, and may feel that they have no voice in the nursing home [55]. Nursing home life may be more stressful for them and, consequently, more destructive to their sense of identity [56]. Together, these factors may have led to low satisfaction and reported QoL, indicating that more attention should be paid to highly educated individuals in care homes to find ways to improve their QoL.

In terms of the most salient findings, older adults with higher levels of optimism, gratitude, and social support had a greater chance of being in the moderate-to-high QoL classes compared to the low QoL groups. The finding is strongly supported by previous studies, which have consistently indicated that higher levels of optimism, gratitude, and social support are associated with greater levels of QoL amongst older adults [14, 22, 25]. Old age is a significant period in which many biological, psychological, and social changes take place, and various challenges are encountered, influencing QoL. Optimism and gratitude both are positive psychological traits which not only focus on or appreciate the positive aspects of life, but can also mobilize affective or cope resources to better deal with and adapt to various challenges with aging [18]. Specifically, optimism can exert a positive effect on QoL via attention, interpretation, and memory cognitive processes [57]. Moreover, optimists are less likely to develop negative emotions, and use more adaptive and active coping strategies when confronting stressful situations than pessimist, which are possible explanations of how optimism positive affects QoL [58].

Regarding gratitude, “grateful” thinking may help individuals concentrate on positive feature of one’s environment, generates positive emotion, and motivates prosocial behavior that helps to build the appropriate social resources to decrease the deleterious effects of adverse events, which is then linked with better QoL [25]. This finding also corresponds to the broaden-and-build theory that greater degree of gratitude is associated with higher QoL [59]. Social support, an important external resource, is mainly provided by family, friends, or significant others, which adds older adults’ social contacts and engagement, promoting their psychological adaptation and successful aging, thereby maintaining good QoL [60]. This finding could be explained by the social escort convoy theory model, in that, individuals are given surrounded from their social networks throughout their lives, these social relationships have a protective function for their health [61]. This finding provides further support to the social support buffer model that social support is a key “buffer” against the negative impact of aging-related stress on QoL [62]. Taken together, these findings highlight the need for establishing and fostering positive psychosocial resources to maintain better QoL among older adults in the context of population aging.

This study had some limitations. Firstly, a cross-sectional design was used, meaning that the results can only identify associations between the different constructs; Additional longitudinal research with a more rigorous study design should be conducted to explore the associations derived from the results of this study. Secondly, data were collected only from older nursing home residents living in Guangzhou; this data may not be representative of the entire elderly Chinese population, limiting the ability to generalize these results to other geographical areas. Lastly, other factors influencing QoL in older adults exist, beyond those assessed in this study; for example, some organizational factors (e.g., staff attitude and care routines in nursing homes) can significantly influence QoL among nursing home residents. These factors should be incorporated in future studies examining the QoL of older adults. Despite these limitations, this study was able to show the benefits of using LPA to identify QoL profiles and examined the role of positive psychosocial resources in influencing QoL levels among older adults living in nursing homes. The results provide new insights into the associations between psychosocial resources and QoL profiles, providing a basis for developing proactive interventions.

## Conclusion

This study examined three distinct profiles of QoL among older adults living in nursing homes using LPA: ‘low QoL with poor psychological health’ class, ‘moderate QoL’ class, and ‘high QoL’ class. Within Chinese older people, we have identified that frequency of weekly physical activity, activity duration, number of chronic diseases, optimism, gratitude, and social support were associated with QoL profiles. Our findings may help staffs in nursing care homes to focus on those residents who are at elevated risk for poor QoL and provide them with targeted and actionable intervention to improve the QoL of older adults in the residential setting.

### Electronic supplementary material

Below is the link to the electronic supplementary material.

Supplementary Material 1

## Data Availability

The datasets used and/or analyzed during the current study available from the corresponding author on reasonable request.

## References

[1] Lunenfeld B, Stratton P (2013). The clinical consequences of an ageing world and preventive strategies. Best Pract Res Clin Obstet Gynaecol.

[2] National Bureau of Statistics of China. The expected goal of sustained recovery and development of the national economy in 2021 has been well achieved[2022-07-01]. Retrieved from: http://www.statsgovcn/xxgk/sjfb/zxfb2020/202201/t20220117_1826436html 2022.

[3] Araujo de Carvalho I, Epping-Jordan J, Pot AM, Kelley E, Toro N, Thiyagarajan JA, Beard JR (2017). Organizing integrated health-care services to meet older people’s needs. Bull World Health Organ.

[4] Cohen JE (2003). Human population: the next half century. Science.

[5] Ćwirlej-Sozańska AB, Sozański B, Wiśniowska-Szurlej A, Wilmowska-Pietruszyńska A (2018). Quality of life and related factors among older people living in rural areas in south-eastern Poland. Ann Agric Environ Med.

[6] Skevington SM, Lotfy M, Fau-O’Connell KA, O’Connell KA (2004). The World Health Organization’s WHOQOL-BREF quality of life assessment: psychometric properties and results of the international field trial. A report from the WHOQOL group. Qual Life Res.

[7] Vincent GK. The next four decades: the older population in the United States: 2010 to 2050(No.1138). US Department of Commerce, Economics and Statistics Administration, US Census Bureau; 2010.

[8] Chatterji S, Byles J, Cutler D, Seeman T, Verdes E (2015). Health, functioning, and disability in older adults–present status and future implications. Lancet.

[9] Stenholm S, Westerlund H, Head J, Hyde M, Kawachi I, Pentti J, Kivimäki M, Vahtera J (2015). Comorbidity and functional trajectories from midlife to old age: the Health and Retirement Study. J Gerontol A.

[10] Lai CK, Leung DD, Kwong EW, Lee RL (2015). Factors associated with the quality of life of nursing home residents in Hong Kong. Int Nurs Rev.

[11] Chin YR, Lee IS, Lee HY (2014). Effects of Hypertension, Diabetes, and/or Cardiovascular Disease on health-related quality of life in elderly Korean individuals: a population-based cross-sectional survey. Asian Nurs Res (Korean Soc Nurs Sci).

[12] Bai W, Zhang J, Smith RD, Cheung T, Su Z, Ng CH, Zhang Q, Xiang YT (2023). Inter-relationship between cognitive performance and depressive symptoms and their association with quality of life in older adults: a network analysis based on the 2017–2018 wave of Chinese longitudinal healthy longevity survey (CLHLS). J Affect Disord.

[13] Elizabeth A, Awick DK, Ehlers S, Aguiñaga, Ana M, Daugherty, Arthur F, Kramer, McAuley E (2017). Effects of a randomized exercise trial on physical activity, psychological distress and quality of life in older adults. Gen Hosp Psychiatry.

[14] Bélanger E, Ahmed T, Vafaei A, Curcio CL, Phillips SP, Zunzunegui MV (2016). Sources of social support associated with health and quality of life: a cross-sectional study among Canadian and latin American older adults. BMJ open.

[15] Seligman ME, Steen TA, Park N, Peterson C (2005). Positive psychology progress: empirical validation of interventions. Am Psychol.

[16] Kim ES, James P, Zevon ES, Trudel-Fitzgerald C, Kubzansky LD, Grodstein F (2019). Optimism and healthy aging in women and men. Am J Epidemiol.

[17] Lara R, Vázquez ML, Ogallar A, Godoy-Izquierdo D. Psychosocial resources for hedonic balance, life satisfaction and happiness in the Elderly: a path analysis. Int J Environ Res Public Health. 2020;17(16). 10.3390/ijerph17165684. 10.3390/ijerph17165684 PMC7459462 32781590

[18] Carver CS, Scheier MF (2014). Dispositional optimism. Trends Cogn Sci.

[19] Shinan-Altman S, Levkovich I, Dror M (2020). Are daily stressors associated with happiness in old age? The contribution of coping resources. Int J Gerontol.

[20] Levkovich I, Shinan-Altman S, Essar Schvartz N, Alperin M (2021). Depression and Health-Related Quality of Life among Elderly patients during the COVID-19 pandemic in Israel: a cross-sectional study. J Prim care Community Health.

[21] Mead JP, Fisher Z, Tree JJ, Wong PTP, Kemp AH (2021). Protectors of Wellbeing during the COVID-19 pandemic: Key roles for Gratitude and tragic optimism in a UK-Based cohort. Front Psychol.

[22] Lee LO, James P, Zevon ES, Kim ES, Trudel-Fitzgerald C, Spiro A 3rd, Grodstein F, Kubzansky LD. Optimism is associated with exceptional longevity in 2 epidemiologic cohorts of men and women. Proc Natl Acad Sci U S A. 2019;116(37):18357–62. 10.1073/pnas.1900712116. 10.1073/pnas.1900712116 PMC6744861 31451635

[23] Craig HJ, Ryan J, Freak-Poli R, Owen A, McNeil J, Woods R, Ward S, Britt C, Gasevic D (2021). Dispositional optimism and all-cause mortality in older adults: a Cohort Study. Psychosom Med.

[24] Wood AM, Froh JJ, Geraghty AW (2010). Gratitude and well-being: a review and theoretical integration. Clin Psychol Rev.

[25] Crouch TA, Verdi EK, Erickson TM (2020). Gratitude is positively associated with quality of life in multiple sclerosis. Rehabil Psychol.

[26] Smedema SM (2020). An analysis of the relationship of character strengths and quality of life in persons with multiple sclerosis. Qual Life Res.

[27] Lou Y, Xu L, Carlsson M, Lan X, Engström M (2022). Quality of life of older people in nursing homes in China-evaluation and application of the Chinese version of the life satisfaction questionnaire. BMC Geriatr.

[28] Maenhout A, Cornelis E, Van de Velde D, Desmet V, Gorus E, Van Malderen L, Vanbosseghem R, De Vriendt P (2020). The relationship between quality of life in a nursing home and personal, organizational, activity-related factors and social satisfaction: a cross-sectional study with multiple linear regression analyses. Aging Ment Health.

[29] Nylund KL, Asparouhov T, Muthén BO (2007). Deciding on the number of classes in latent class analysis and growth mixture modeling: a Monte Carlo simulation study. Struct Equation Modeling: Multidisciplinary J.

[30] Kongsted A, Nielsen AM (2017). Latent class analysis in health research. J Physiother.

[31] Spurk D, Hirschi A, Wang M, Valero D, Kauffeld S (2020). Latent profile analysis: a review and how to guide of its application within vocational behavior research. J Vocat Behav.

[32] Choi EH, Kang MJ, Lee HJ, Yun MS. A latent class analysis of Health-related quality of life in Korean older adults. Int J Environ Res Public Health. 2021;18(15). 10.3390/ijerph18157874. 10.3390/ijerph18157874 PMC8345710 34360166

[33] Băjenaru L, Balog A, Dobre C, Drăghici R, Prada GI (2022). Latent profile analysis for quality of life in older patients. BMC Geriatr.

[34] Statistical Bulletin on National Economic and Social Development of the People’s Republic of China. National Bureau of Statistics of China; Beijing, China. 2022. Retrieved from http://www.statsgovcn/sj/zxfb/202302/t20230228_1919011html.

[35] de Medeiros MMD, Carletti TM, Magno MB, Maia LC, Cavalcanti YW, Rodrigues-Garcia RCM (2020). Does the institutionalization influence elderly’s quality of life? A systematic review and meta-analysis. BMC Geriatr.

[36] Liu C, Luo D, Zhou Y, Zhang G, Feng X, Wang Z, Chen J, Bi Q (2022). Optimism and subjective well-being in nursing home older adults: the mediating roles of gratitude and social support. Geriatr Nurs.

[37] Chow SC, Shao J, Wang H, Lokhnygina Y. Sample size calculations in clinical research[M]. CRC press; 2017.

[38] Masnoon NA-O, Shakib S, Kalisch-Ellett L, Caughey GE (2017). What is polypharmacy? A systematic review of definitions. BMC Geriatr.

[39] Xia P, Li N, Hau KT, Liu C, Lu Y (2012). Quality of life of Chinese urban community residents: a psychometric study of the mainland Chinese version of the WHOQOL-BREF. BMC Med Res Methodol.

[40] Min SK, Kim KI, Lee CI, Jung YC, Suh SY, Kim DK (2002). Development of the Korean versions of WHO Quality of Life scale and WHOQOL-BREF. Qual Life Res.

[41] Scheier MF, Carver CS, Bridges MW (1994). Distinguishing optimism from neuroticism (and trait anxiety, self-mastery, and self-esteem): a reevaluation of the Life Orientation Test. J Personality Social Psychol.

[42] Lai JCL, Yue X (2000). Measuring optimism in Hong Kong and mainland Chinese with the revised life orientation test. Pers Indiv Differ.

[43] McCullough ME, Emmons RA, Tsang J-A (2002). The grateful disposition: a conceptual and empirical topography. J Personality Social Psychol.

[44] Wei C, Wu HT, Kong XN, Wang H (2011). Revision of Gratitude Questionnaire-6 in Chinese adolescent and its validity and reliability. Chin J Sch Health.

[45] Zimet GD, Powell SS, Farley GK, Werkman S, Berkoff KA (1990). Psychometric characteristics of the Multidimensional Scale of Perceived Social Support. J Pers Assess.

[46] Jiang QJ. Perceived social support scale. Chin J Behavioral Med Sci, 2001:41–2.

[47] Kenneth P, Burnham DR, Anderson (2004). Multimodel inference: understanding AIC and BIC in model selection. Sociol Methods.

[48] Celeux G, Soromenho G (1996). An entropy criterion for assessing the number of clusters in a mixture model. J Classif.

[49] Nagin DS (2005). Posterior group-membership probabilities. Group-based modeling of Development Cambridge.

[50] Lamberti N, Manfredini F, Babjaková J, Gallè F, Medijainen K, Karatzaferi C, Pavlova I, Netz Y, López-Soto PJ (2022). Effect of physical activity interventions on quality of life in older adults: a protocol for systematic review and meta-analysis. Medicine.

[51] Fiona C, Bull SS, Al-Ansari S, Biddle K, Borodulin MP, Buman G, Cardon C, Carty J-P, Chaput (2020). Sebastien Chastin, Roger Chou. World Health Organization 2020 guidelines on physical activity and sedentary behaviour. Br J Sports Med.

[52] You Y, Teng W, Wang J, Ma G, Ma A, Wang J, Liu P (2018). Hypertension and physical activity in middle-aged and older adults in China. Sci Rep.

[53] Marquez DX, Aguiñaga S, Vásquez PM, Conroy DE, Erickson KI, Hillman C, Stillman CM, Ballard RM, Sheppard BB, Petruzzello SJ, King AC, Powell KE (2020). A systematic review of physical activity and quality of life and well-being. Transl Behav Med.

[54] Buckinx F, Mouton A, Reginster JY, Croisier JL, Dardenne N, Beaudart C, Nelis J, Lambert E, Appelboom G, Bruyère O (2017). Relationship between ambulatory physical activity assessed by activity trackers and physical frailty among nursing home residents. Gait Posture.

[55] Inge Renne, Gobbens RJ. Effects of frailty and chronic Diseases on quality of life in Dutch community-dwelling older adults: a cross-sectional study. Clin Interv Aging 2018:325–34. 10.2147/CIA.S156116. 10.2147/CIA.S156116 PMC5833750 29520132

[56] Van Malderen L, De Vriendt P, Mets T, Gorus E (2016). Active ageing within the nursing home: a study in Flanders, Belgium. Eur J Ageing.

[57] Diener E, Lucas RE, Oishi S. Advances and open questions in the science of subjective well-being. Collabra Psychol. 2018;4(1). 10.1525/collabra.115. 10.1525/collabra.115 PMC6329388 30637366

[58] Schou I, Ekeberg Ø, Ruland CM (2005). The mediating role of appraisal and coping in the relationship between optimism-pessimism and quality of life. Psychooncology.

[59] Fredrickson BL (2001). The role of positive emotions in positive psychology: the broaden-and-build theory of positive emotions. Am Psychol.

[60] Lestari SK, Luna Xd, Eriksson M, Malmberg G, Ng N (2021). A longitudinal study on social support, social participation, and older europeans’ quality of life. SSM-Population Health.

[61] Antonucci TC, Ajrouch KJ, Birditt KS (2014). The convoy model: explaining social relations from a multidisciplinary perspective. Gerontologist.

[62] Karen DL (2000). Social support, negative social interactions, and psychological well-being. Social Service Review.

