# Correction: Quality of life profiles and its association with predictors amongst Chinese older adults in nursing homes: a latent profile analysis

**DOI:** 10.1186/s12877-023-04496-8

**Published:** 2024-01-02

**Authors:** Chunqin Liu, Qing Luo, Dongyi Luo, Ying Zhou, Xue Feng, Zihan Wang, Jiajian Xiao, Qiulin Bi, Graeme Drummond Smith

**Affiliations:** 1 School of Nursing, Guangzhou Medical University, Guangzhou, Guangdong 510182 China; 2 School of Medicine, Jinggang Shan University, Jian, Jiangxi 343009 China; 3 School of Heath Industry, Guangdong Open University, Zhongshan, Guangdong 528499 China; 4 Finance Division of Guangzhou Medical University, Guangzhou, Guangdong 511436 China; 5 Guangzhou Songhe Nursing Home, Guangzhou, Guangdong 510250 China; 6 School of Health Sciences, Caritas Institute of Higher Education, Hong Kong SAR, 999077 China

**BMC Geriatrics (2023) 23:740**

10.1186/s12877-023-04456-2

After publication of this article [1], the authors reported that in this article the last sentence of the section ‘Latent profile analysis for QoL’ (‘a, b, c showed the results of the Bonferroni post-hoc tests and there is a statistically significant between two. e.g. for physical 'a<b, c' means that class 1 has a mean score that is lower than class 2 and 3’) should be placed as a table note below Table 2.

