# Supplementary material for: Quality of life profiles and its association with predictors amongst Chinese older adults in nursing homes: a latent profile analysis
Source: BMC Geriatr. 2023 Nov 14;23:740. doi: 10.1186/s12877-023-04456-2 (PMC10644486; doi:10.1186/s12877-023-04456-2)
Supplement: Supplementary file 1 — Supplementary Material 1 [file 12877_2023_4456_MOESM1_ESM.doc]

**Table S1 Three nursing homes in demographic, psychosocial characteristics and QoL profiles among older adults(n=354)**

| Variables | Nursing home 1  *n* (%)  121 (34.2) | Nursing home 2  *n* (%)  92(26.0) | Nursing home 3  *n* (%)  141(39.8) | *χ^2^/F* | *P* |
| --- | --- | --- | --- | --- | --- |
| Age(years) M(SD) | 84.5(6.8) | 86.6(6.3) | 86.6(6.2) | 2.827 | 0.061 |
| Gender  Female  Male | 83(68.6)  38(31.4) | 70(76.0)  22(24.0) | 111(78.7)  30(21.2) | 3.462 | 0.177 |
| Nursing home location (Urban) | 121(100) | 92(100) | 141(100) | *-* | *-* |
| Nursing home ownership  (Privately owned) | 121(100) | 92(100) | 141(100) | *-* | *-* |
| Bed capacity (300 and over beds) | 121(100) | 92(100) | 141(100) | - | - |
| Medical-nursing combined model (Yes) | 121(100) | 92(100) | 141(100) | - | - |
| Provided various daily life activities (Yes) | 121(100) | 92(100) | 141(100) | - | - |
| QoL subscales: M(SD)  Physical health | 58.6(16.7) | 59.2(15.6) | 62.9(13.8) | 3.011 | 0.051 |
| Psychological health | 58.7(16.4) | 56.5(13.3) | 60.3(12.4) | 1.552 | 0.213 |
| Social relationships | 58.9(13.9) | 56.5(11.5) | 58.5(10.2) | 1.653 | 0.193 |
| Environmental health | 62.7(14.5) | 61.9(12.2) | 61.1(10.2) | 0.589 | 0.556 |
| QoL profiles  Class1  Class2  Class3 | 24(19.8)  52(43.0)  45(37.2) | 16(17.4)  51(55.4)  25(27.2) | 24(17.0)  60(42.6)  57(40.4) | 5.509 | 0.239 |

Note: Class1= Low QoL with poor psychological health, Class2=Moderate QoL, Class3=High QoL.

**Table S2 Correlation analyses between the QoL subscales, optimism, gratitude, and social support.**

| Variables | 1 | 2 | 3 | 4 | 5 | 6 | 7 |
| --- | --- | --- | --- | --- | --- | --- | --- |
| 1 Physical health | 1 |  |  |  |  |  |  |
| 2 Psychological health | 0.70* | 1 |  |  |  |  |  |
| 3 Social relationships | 0.56* | 0.60* | 1 |  |  |  |  |
| 4 Environmental health | 0.68* | 0.75* | 0.56* | 1 |  |  |  |
| 5 Optimism | 0.35* | 0.42* | 0.32* | 0.35* | 1 |  |  |
| 6 Gratitude | 0.22* | 0.32* | 0.30* | 0.33* | 0.28* | 1 |  |
| 7 Social support | 0.30* | 0.48* | 0.49* | 0.40* | 0.29* | 0.49* | 1 |

Note: **P*<0.01.
